# Evolving Trends in Autoimmune Hepatitis Research: A Bibliometric Analysis From 2004 to 2024

**DOI:** 10.1111/jcmm.71348

**Published:** 2026-09-02

**Authors:** Yuling Kong, Zhifeng Lin

**Affiliations:** ^1^ Department of Hepatology, Department of Medical Record, Guangdong Provincial Key Laboratory of Major Obstetric Diseases, Guangdong Provincial Clinical Research Center for Obstetrics and Gynecology The Third Affiliated Hospital, Guangzhou Medical University Guangzhou China

Dear Editor,

Autoimmune hepatitis (AIH) is a heterogeneous, immune‐mediated liver disease with diverse clinical presentations. Its diagnosis relies on combined clinical, biochemical, serological and histological findings rather than a single marker [1, 2]. Corticosteroid‐based immunosuppression remains the cornerstone of treatment, yet diagnostic uncertainty, relapse and treatment toxicity persist [1, 2]. We therefore examined whether the recent research portfolio is shifting towards questions that could address these uncertainties, rather than merely increasing in volume.

We analysed English‐language articles and reviews indexed in the Web of Science Core Collection between 1 January 2004 and 31 December 2024. The search and screening were completed on 19 April 2025. We used VOSviewer 1.6.20, CiteSpace 6.4.R1 and the R package bibliometrix to analyse collaboration, co‐citation, keyword co‐occurrence, citation bursts and thematic evolution [3, 4, 5].

The 6310 records spanned 110 countries, 5717 institutions, 28,429 authors and 1348 journals, with annual output rising markedly after 2020 (Figure 1). The United States, China, the United Kingdom and Japan were the leading contributors. Mayo Clinic and King's College Hospital London occupied prominent positions in the collaboration network. Liver International published the most records, whereas Hepatology was the most frequently co‐cited journal. Albert J. Czaja and Ansgar W. Lohse were prominent in the authorship and citation analyses. These rankings reflect research activity and network position, not methodological quality or clinical impact.

**FIGURE 1:**
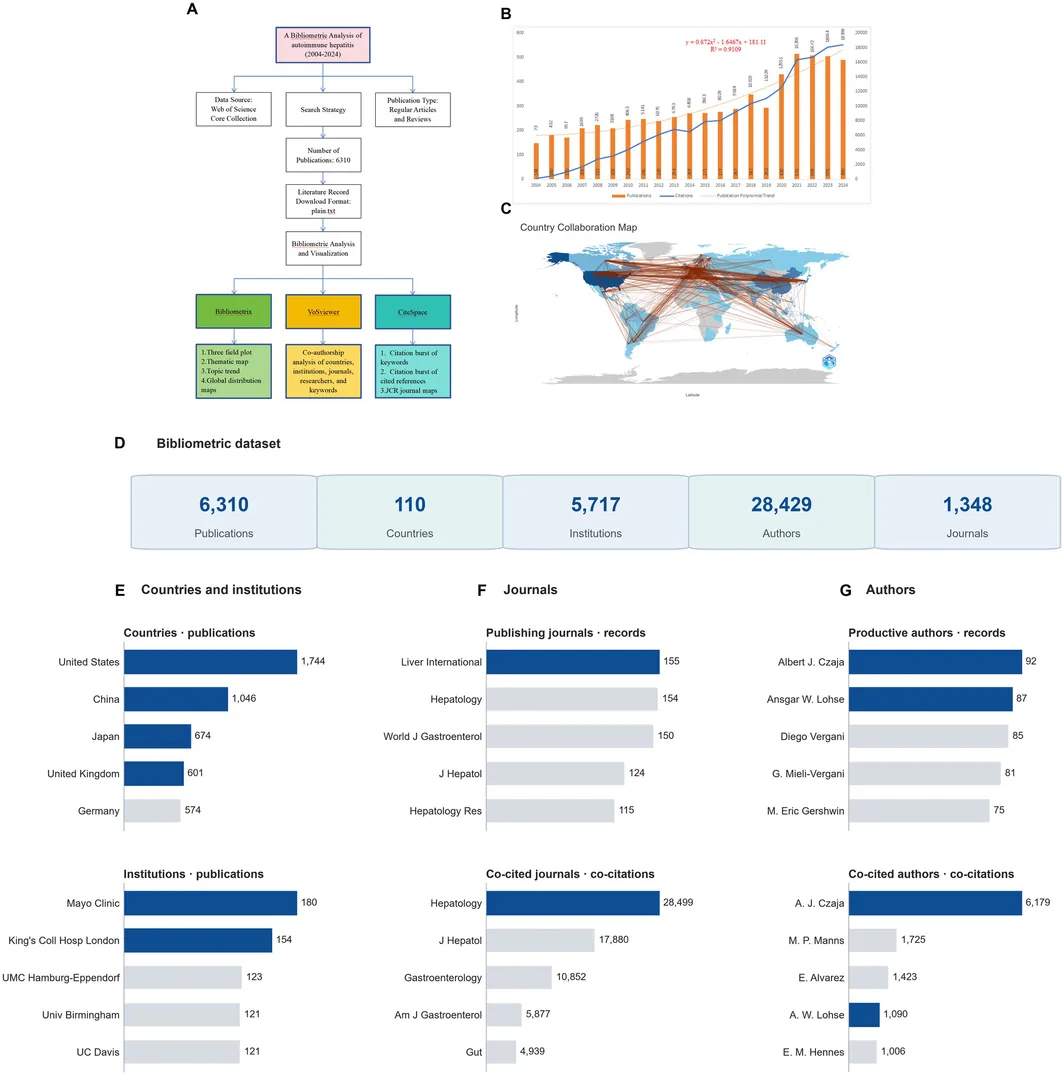
Study workflow, publication growth and leading contributors in AIH research. (A) Flow diagram of literature retrieval, record screening and bibliometric analysis. (B) Annual publication and citation output, together with the fitted publication trend, showing a marked increase in research activity after 2020. (C) Country‐level international collaboration network. (D) Overall composition of the dataset. (E) Five leading countries and institutions ranked by publication count. (F) Five most productive journals ranked by record count and five most frequently co‐cited journals ranked by co‐citation count. (G) Five most productive authors ranked by record count and five most frequently co‐cited authors ranked by co‐citation count.

More important than growth alone was the shift in the questions attracting attention. Earlier research focused on diagnosis, immunosuppressive treatment and liver transplantation. Recent burst and trend analyses increasingly highlighted the gut microbiota, gut–liver axis, biomarker discovery, immune regulation, multi‐omics and computational approaches (Figure 2). In a clinical cohort, Liwinski et al. identified reduced microbial diversity and a disease‐specific decline in Bifidobacterium [6]. Lower abundance was associated with failure to achieve remission [6]. Zhang et al. reported increased intestinal permeability among patients with AIH. In murine models, barrier dysfunction and bacterial translocation intensified RIP3‐associated hepatic inflammation, while antibiotic treatment attenuated liver injury [7]. Together, these findings support further investigation of host–microbe interactions in AIH. However, human associations and animal interventions do not establish a causal gut mechanism in patients.

**FIGURE 2:**
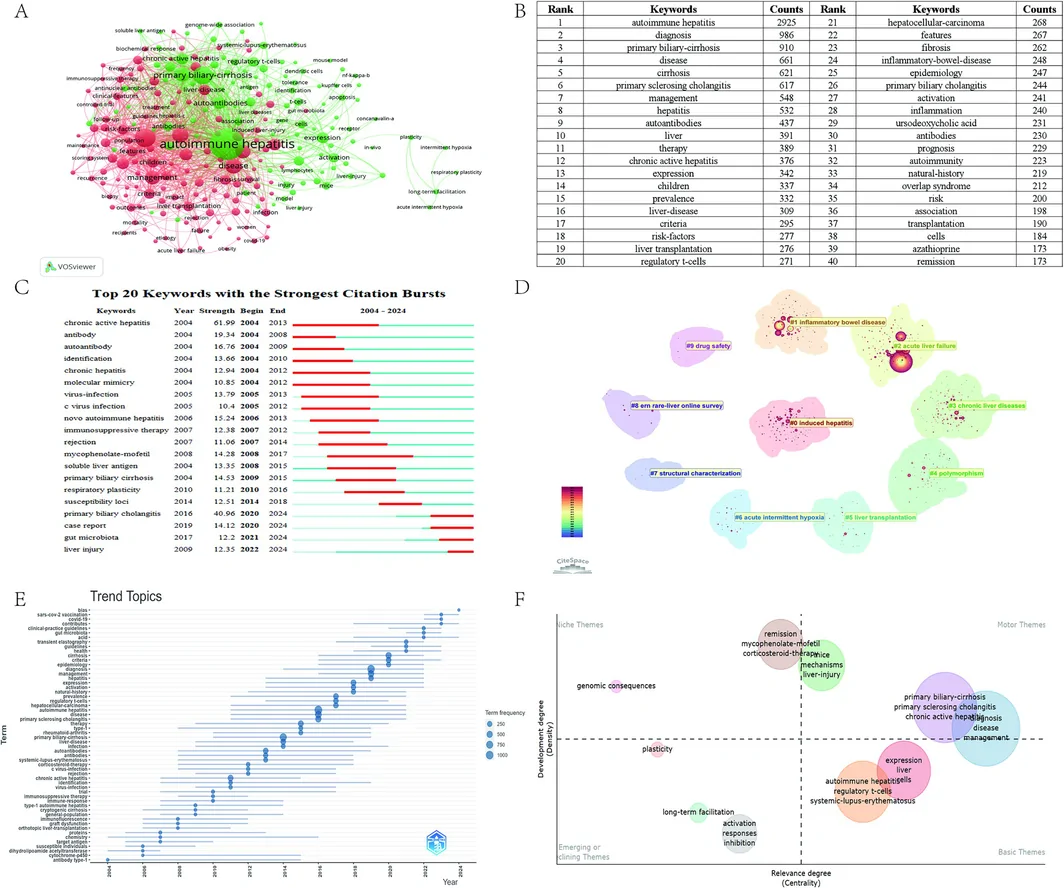
Keyword structure and topic evolution in autoimmune hepatitis research. (A) Keyword co‐occurrence network. (B) Forty most frequent keywords. (C) Twenty strongest keyword citation bursts. (D) CiteSpace keyword clusters. (E) Trend topics. (F) Thematic map.

Biomarker research warrants similarly cautious interpretation of the available evidence. A review catalogued candidate gene‐expression, protein, metabolite and immune‐cell markers, while emphasising that clinically useful predictors remain inadequately established [8]. Primary studies identified cirrhosis‐associated serum metabolite signatures [9], whole‐blood transcriptional differences and candidate fibrosis‐linked genes [10], and differential protein abundance in a paediatric cohort [11]. These findings represent candidate signals rather than validated clinical tests. Current computational studies should likewise be regarded as preliminary evidence. One deep‐learning analysis included 123 pretreatment biopsies [12], while an exploratory machine‐learning model used 233 development patients and a 33‐patient validation cohort [13]. Limited cohort sizes, single‐centre settings and retrospective designs constrain the transportability of both approaches.

Bibliometric analyses reveal research attention, knowledge structures and directional change, but they cannot establish biological causality, diagnostic performance or treatment effectiveness. Publication counts, centrality and co‐citation measures scholarly communication. They should not serve as proxies for study quality or patient benefit.

Our analysis indicates that AIH research is shifting towards microbiome, biomarker and computational approaches, but these priorities remain exploratory and require coordinated validation. Multicentre prospective studies should integrate standardised clinical phenotypes, treatment exposure, histology and outcomes with immunomic, microbiome and multi‐omics data. Sampling and analyses should be prespecified, with candidate mechanisms and biomarkers validated independently and tested experimentally where appropriate. Shared standards would help distinguish reproducible biology from transient hotspots and translate robust signals into clinically useful tools.

## Author Contributions

**Yuling Kong:** writing – original draft, methodology, validation, software, formal analysis, data curation. **Zhifeng Lin:** conceptualization, investigation, writing – review and editing, visualization, validation, methodology, supervision.

## Funding

The authors have nothing to report.

## Ethics Statement

The authors have nothing to report.

## Consent

The authors have nothing to report.

## Conflicts of Interest

The authors declare no conflicts of interest.

## Data Availability

The data that support the findings of this study are available from the corresponding author upon reasonable request.
